# On the Change of Measure for Brownian Processes

**DOI:** 10.3390/e27060594

**Published:** 2025-05-31

**Authors:** Francis J. Pinski

**Affiliations:** Department of Physics, University of Cincinnati, Cincinnati, OH 45221, USA; frank.pinski@uc.edu; Tel.: +1-513-432-8717

**Keywords:** stochastic differential equations, Brownian dynamics, singular limit, change of measure

## Abstract

Sometimes, limits can be singular, implying that they take on different values depending on the order of arithmetic operations. In other words, the limit map lacks commutativity. While all such limits are mathematically valid, only one can be the physical limit. The change of measure for Brownian processes illustrates this phenomenon. A substantial body of elegant mathematics centered around continuous-time Brownian processes has been embraced by the physics community to investigate the nonequilibrium and equilibrium thermodynamics of systems composed of atoms and molecules. In this paper, we derive the continuous-time limit of discrete-time Brownian dynamics, specifically focusing on the change of measure. We demonstrate that this result yields the physical limit that differs from the commonly used expression. Consequently, the concepts of “the most probable path”, “minimum thermodynamic action”, and “the small-noise limit” are unphysical mathematical artifacts.

## 1. Introduction

Some observations of the movement of a particle show that it seems to move in a zig-zag pattern, as if driven by random, thermal forces. This “Brownian” motion is named after Robert Brown [1], who observed this behavior in 1827. The mathematical description of the thermal motion of a particle embedded in an external potential and buffeted by random forces is described by a stochastic differential equation (SDE), the overdamped Langevin equation, often referred to as Brownian dynamics. The mathematical basis was initiated by Wiener [2,3] in the 1920s. In the ensuing century, many textbooks have been written covering the mathematical developments; as an example, see Reference [4].

Among the many theorems that have been developed over the last century, the ones regarding the continuous-time process stand out as being particularly important. In particular, the Itô–Girsanov expression for the Radon–Nikodym derivative is the basis for the equivalency of measures associated with continuous-time Brownian processes with differing deterministic forces.

Here, we re-examine the change of measure, not disputing the major theorems mentioned above. Instead of considering continuous-time Brownian processes, we start with interpreting the discrete-time realization of the continuous-time process as the physical picture and then consider the continuous-time limit of such processes. One might ask, why bother? Often, it is illuminating to numerically construct particle motion as it moves from one free-energy basin to another to uncover transition pathways. In computer algorithms designed to sample such paths, time advances in discrete steps. As one approaches the continuous-time limit, one encounters divergent results for various properties. To alleviate this problem by isolating the singularity, a quantity called the “change of measure” was introduced. As we show below, the formula for the change of measure depends on the route one takes to evaluate the continuous-time limit.

Historically, the first route to consider was forming the change of measure for continuous-time processes. However, when following this route, unphysical features have emerged in computer calculations. For instance, Malsom and Pinski [5,6,7] used the Itô–Girsanov expression to examine paths for a particle moving in a one-dimensional potential with two degenerate wells, one of which was significantly broader than the other. In these calculations, they found that the paths indicated that the particle would spend most of its time in the narrow well rather than the broad well. This unphysical result was directly attributed to the “Laplacian” term in the Itô–Girsanov expression. In a more recent paper [7], the author analyzed doubly pinned paths for a particle traversing a two-dimensional potential landscape. This potential featured two distinct free-energy wells connected by a very narrow channel devoid of an energy barrier. Using the Itô–Girsanov (IG) expression, the sampled paths indicated that the particle gets stuck in the narrow channel. This unphysical result was traced to the substantial value of the Laplacian term along the channel. It is worth noting that both computational algorithms found sensible, physical paths when using the discrete-time form of the Onsager–Machlup (OM) functional and the same numerical parameters.

The above then motivates a different way to determine the continuous-time limit, which is to evaluate the change of measure for discrete-time processes before taking the continuous-time limit. This alternative approach is what we investigate here. Both limiting procedures are mathematically correct; however, as we show below, the latter one provides the physical limit. In this physical limit, many of the commonly used concepts, such as the MPP [8] (most probable path), do not exist.

This paper is organized as follows. In Section 2, we specify the notation used, and define the “common-noise” and “common-path” constructions. In this section, we also calculate the expression for the change of measure between two processes, and the concomitant the Kullback–Leibler (KL) divergence for each of the two constructions. In Section 3, we present our main result: the physical continuous-time limit of the change of measure between two Brownian processes is given by the common-noise construction. We continue with a discussion of the results in Section 4, where we explore some numerical issues and the concept of “thermodynamic action”. Finally, in Section 5, we end with a summary and additional ramifications of the current work.

## 2. Model

In this section, for both continuous and discrete time, we define the stochastic (Brownian) processes in terms of mathematical equations. Then, we proceed to examine the “common-noise” and “common-path” constructions and produce expressions for the change of measure and KL divergence between two processes. Although this paper examines a single spatial dimension, the conclusions derived herein are readily extendable to higher dimensions.

### 2.1. Preliminaries

Consider the concrete example of a particle moving in one spatial dimension under the influence of an external potential, $U_{\alpha }\left(x\right)$ at a temperature ϵ. The motion is described by Brownian dynamics; namely, the stochastic differential equation provides the position *x* as a function of time *t*:

(1) $$dx_{t}\;=\;f_{\alpha }\left(x_{t}\right)\;dt\;+\;\sqrt{2\;\epsilon \;}\;dW_{t}\;,$$

with $f_{\alpha }\left(x\right)=-\;\partial U_{\alpha }/\partial x$ and $dW_{t}$ being the standard Wiener process. For a nonzero time step, Δt>0, the Euler–Maruyama expression [9] is

(2) $$x_{i}-x_{i-1}\;=\;f_{\alpha }\left(x_{i-1}\right)\;\Delta t\;+\;\sqrt{2\;\epsilon \;\Delta t\;}\;\xi_{i}\;,$$

where the index *i* is related to the time t=iΔt, and $\xi_{i}$ are Gaussian distributed random variates with mean zero and unit variance. To be consistent with thermodynamics, the external potential $U_{\alpha }\left(x\right)$ must belong to the Kato class [10], which includes smooth functions bounded from below.

### 2.2. The Physical Model

Usually, Equation (2) is considered as an approximation to Equation (1). Here, we attach meaning to Equation (2) and use it to represent a physical model. The system of interest is connected to a thermal reservoir, kept at a temperature ϵ. This reservoir is the source of the noise. The noise is Gaussian-distributed with mean zero and unit variance. We define the **noise history** of any path with $N_{t}$ time steps as the sequence $N=\{\xi_{i}\}$; the sequence N has $N_{t}$ elements.

Unless explicitly stated, we will be working with paths described in $R^{N}$, where $N=N_{t}+1$ and $N_{t}$ is the number of time steps. In addition, the noise is due to an external thermal reservoir. Thus, the noise is independent of the system being studied; the noise and the particle positions are not correlated. The path positions for a single trajectory may be highly correlated with the noise. However, for a point $x_{P}$ and an ensemble of paths each containing $x_{P}$, the value of the noise acting at $x_{P}$ will be Gaussian-distributed.

For a path of (time) length *T*, with $N_{t}\;\Delta t\;=\;T\;$, the probability density (Lebesgue measure) of the noise history, $\left\{\xi_{i}\right\}$, is given by

(3) $$P_{N_{t}}\;\left(\left\{\xi_{i}\right\}\right)\;\prod_{i=1}^{N_{t}}\;d\xi_{i}={\left(2\;\pi \right)}^{-N_{t}/2}\prod_{i=1}^{N_{t}}\left(\exp\;(-\frac{1}{2}\xi_{i}^{2}\;)\;d\xi_{i}\;\right)\;.$$

The probability density can be expressed in terms of the path positions, $P_{N_{t}}^{OM}\left(\left\{x_{i}\right\}\right)$, and given by

(4) $$P_{N_{t}}^{OM}\;\left(\left\{x_{i}\right\}\right)\;\prod_{i=1}^{N_{t}}dx_{i}=\;{\left(4\;\epsilon \;\Delta t\;\pi \right)}^{-N_{t}/2}\exp\;\left(-I_{N_{t},\;\alpha }\left(\left\{x_{i}\right\}\right)\right)\prod_{i=1}^{N_{t}}dx_{i}$$

where $I_{N_{t},\;\alpha }\left(\left\{x_{i}\right\}\right)$ is the Onsager–Machlup (OM) functional [11] and is given by

(5) $$I_{N_{t},\;\alpha }\left(\left\{x_{i}\right\}\right)=\;\frac{\Delta t}{4\;\epsilon }\;\sum_{i=1}^{N_{t}}{\left(\;\frac{x_{i}-x_{i-1}}{\Delta t}\;-\;f_{\alpha }\left(x_{i-1}\right)\right)}^{2}\;.$$

Here, we again emphasize that the path density is determined by the noise, Equation (3) or (4), which is emanating from a heat reservoir. The noise is Gaussian-distributed and is independent of the system being studied. Since

(6) $$\xi_{i}=\sqrt{\frac{\Delta t}{2\;\epsilon }\;}\;\left(\;\frac{x_{i}-x_{i-1}}{\Delta t}\;-\;f_{\alpha }\left(x_{i-1}\right)\right)\;,$$

the OM functional is also Gaussian-distributed with mean and variance $N_{t}/2$. It is the multiplicity of paths that determines the optimal pathways for a particle to move from one free-energy basin to another.

To better grasp this concept, visualize a pair of standard, balanced six-sided dice, one black and the other red. Each individual throw yields an outcome with a one-in-thirty-six probability of occurring. The most probable sum of their faces is seven, as it emerges most frequently from the thirty-six possible outcomes. Similarly, the noise generated by the heat reservoir is represented by a sequence of independent and identically distributed (iid) Gaussian random variables. It is not the probabilities of individual paths that determine the most probable pathways between two configurations, but rather the multiplicity, the sheer number of paths.

### 2.3. Common-Noise History

**Figure 1:**
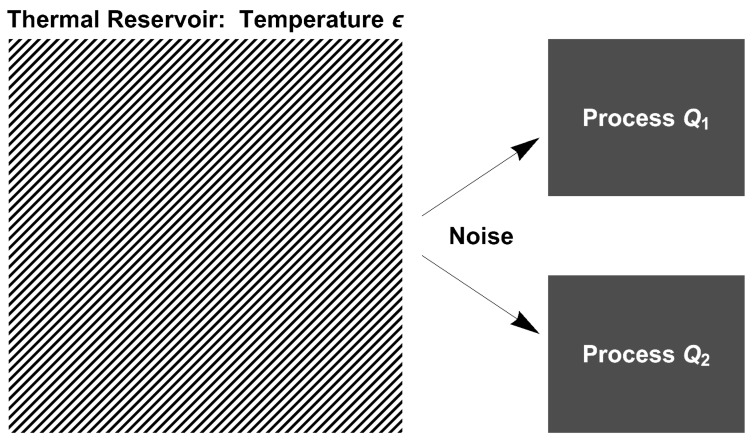
Schematic representation of the thought experiment. Two discrete-time processes, $Q_{1}$ and $Q_{2}$, are connected to a single thermal heat reservoir. The heat reservoir, kept at a temperature ϵ, is the source of the noise, the stochastic force that a particle experiences. In this experiment, both processes experience the same noise histories. The stochastic forces in both processes are given by the same sequence of iid Gaussian random variates.

We have now laid the groundwork to understand how the measure changes from one Brownian process to another. By examining Equation (3), it becomes evident that the measure is independent of the force (the drift). Consequently, for discrete time, two Brownian measures are identically Gaussian. The underlying physical concept behind this mathematical statement is that the noise is a characteristic of the external heat bath and is independent of the system being studied. This idea is further reinforced by considering the following thought experiment. Connect a constant-temperature heat reservoir to two (or more) separated systems, ensuring that all the systems receive the same noise input, as schematically shown in Figure 1. Despite being subjected to the same noise history, distinct Brownian processes generate different paths. However, the measure of each of the processes corresponds to the same Gaussian measure, and thus such paths are connected through a bijective map.

To make the above ideas concrete, consider the two discrete-time processes:

(7) $$Q_{1}:\;\;dx_{t}\;=\;f_{1}\left(x_{t}\right)\;dt\;+\;\sqrt{2\;\epsilon \;}\;dW_{t}\;\;\;\mathrm{and}$$

(8) $$\;Q_{2}:\;\;dz_{t}\;=\;f_{2}\left(z_{t}\right)\;dt\;+\;\sqrt{2\;\epsilon \;}\;dW_{t}\;.\;\;$$

We use the equations for the discrete-time process given by Equation (2). Both processes use identical values for the (time) length, *T*, the time increment Δt, and the temperature, ϵ. We assume that the starting points $x_{0}$ and $z_{0}$ are both zero. If one uses the same noise history $N=\{\xi_{i}\}$, one would find that the ending positions differ; $x_{T}\neq z_{T}$, almost surely, as long as $f_{1}$ and $f_{2}$ differ.

Looking at Equation (3), the path probability only depends on the noise history, the sequence N. Clearly, measures for the discrete-time processes, $Q_{1}$ and $Q_{2}$, are identical.

### 2.4. Common-Noise Construction Revisited

Typically, when one considers the processes $Q_{1}$ and $Q_{2}$, one generates paths and considers them instead of the noise histories. Consider a path $Z=\{z_{i}\}$ generated by process $Q_{2}$ by a noise history $N_{2}=\left\{\xi_{i}^{\left(2\right)}\right\}$, and a path $X=\{x_{i}\}$ generated by process $Q_{1}$ by a noise $N_{1}=\left\{\xi_{i}^{\left(1\right)}\right\}$. The set of all possible paths *X* is identical to the set of all possible paths *Z*. Consider a particular path *Z* generated by $Q_{2}$ by the noise history $N_{2}$. Using this same noise history, the path $\tilde{X}=\left\{{\tilde{x}}_{i}\right\}$ is generated by $Q_{1}$. This then defines the bijective map, *B*, namely $\tilde{X}=B\left(Z\right)$. The change of measure, the reweighting, L(Z), of the probabilities, is given by

(9) $$L\left(Z\right)=\frac{Q_{1}}{Q_{2}}=\exp\;\left(I_{N_{t},\;2}\left(\left\{{\tilde{x}}_{i}\right\}\right)-I_{N_{t},\;1}\left(\left\{z_{i}\right\}\right)\right)=1.$$

Equation (9) is simply another a restatement of the physical idea expressed in Section 2.3, and leads to the vanishing of the Kullback–Leibler (KL) divergence. From the change of measure, one can obtain the Kullback–Leibler (KL) divergence, given by considering

(10) $$\begin{array}{c} D_{KL}(Q_{2}\;||\;Q_{1})=\;-E_{2}\;\ln\frac{Q_{2}}{Q_{1}}\;=\;0. \end{array}$$

Clearly, the KL divergence is zero since each term in Equation (10) vanishes. We note the importance of the bijective map *B*. The same noise history $N_{2}$ generates *Z* and $\tilde{X}$, using the processes $Q_{2}$ and $Q_{1}$, respectively.

Consistent with the results of the previous section, the KL divergence vanishes, indicating that the measures of two Brownian processes are equivalent. This manner of evaluating the KL divergence will be denoted as the “common-noise construction”.

### 2.5. Common-Path Construction

Instead of using a common noise history, one can construct the ratio $Q_{1}/Q_{2}$ using identical paths. The Equations (7) and (8) must be modified since the Wiener processes are different in the two SDEs. A path $Z=\{z_{i}\}$ can be generated by process $Q_{2}$ using the noise $N_{2}$. The same path *Z* can be generated by the process $Q_{1}$ using a different noise $N_{1}$. We can calculate the common-path construction for the Kullback–Leibler (KL) divergence:

(11) $$\begin{array}{c} {\tilde{D}}_{KL}\;(Q_{2}\;||\;Q_{1})=E_{2}\left(\;I_{N_{t},\;2}\left(\left\{z_{i}\right\}\right)\;-\;I_{N_{t},\;1}\left(\left\{z_{i}\right\}\right)\right). \end{array}$$

In Equation (11), the far right-hand-side term depends on the difference $I_{N_{t},\;2}\left(\left\{z_{i}\right\}\right)-I_{N_{t},\;1}\left(\left\{z_{i}\right\}\right)$; some terms cancel in this difference. We then define J eliminating these canceling terms; namely,

(12) $$\begin{array}{c} J_{N_{t},\;\alpha }\left(Z\right)=\;\frac{\Delta t}{2\;\epsilon }\sum_{i=1}^{N_{t}}\;\left(\frac{1}{2}\;f_{\alpha }^{2}\left(z_{i-1}\right)-\left(\frac{z_{i}-z_{i-1}}{\Delta t}\right)\;f_{\alpha }\left(z_{i-1}\right)\right). \end{array}$$

And the KL divergence becomes

(13) $$\begin{array}{c} {\tilde{D}}_{KL}\;(Q_{2}\;||\;Q_{1})=\;-E_{2}\;\left(J_{N_{t},\;1}\left(Z\right)\;-\;J_{N_{t},\;2}\left(Z\right)\right)\;, \end{array}$$

where *Z* is a realization of the path generated by process $Q_{2}$. This cancelation eliminates the possibly of a singularity in the KL divergence due to I (see Equation (4)) when evaluating the continuous-time limit.

We can now take the continuous-time (CT) limit (Δt→0) of $J_{N_{t},\;\alpha }\left(Z\right)$ by applying the integration-by-parts formula from Itô calculus. See Appendix B for details. The resulting expression becomes

(14) $$\begin{array}{c} J_{\alpha ;\;T}^{CT}\left(Z\right)\;=\;\;\lim_{\Delta t\to \;0}J_{N_{t},\;\alpha }\left(\left\{z_{i}\right\}\right)=\frac{1}{2\;\epsilon }\;(U_{\alpha }\left(z_{T}\right)-U_{\alpha }\left(z_{0}\right))\\ \;\;+\;\;\frac{1}{2\;\epsilon }\int_{0}^{T}\;\;\;dt\;\left(\;\frac{1}{2}\;f_{\alpha }^{2}\left(z_{t}\right)-\;\epsilon \;U_{\alpha }^{\prime \prime }\left(z_{t}\right)\right)\;. \end{array}$$

The KL divergence within the common-path construction is given by

(15) $${\tilde{D}}_{KL}^{CT}(Q_{2}\left|\right|Q_{1})\;=\;E_{2}\left(J_{1;\;,T}^{CT}\left(Z\right)-J_{2;\;T}^{CT}\left(Z\right)\right)\;.$$

In contrast, the above result differs from the continuous limit of the KL divergence within the common-noise construction as within that construction $D_{KL}(Q_{2}\left|\right|Q_{1})\;=0$ for any non-negative Δt, no matter how small it is.

Note that Equations (13) and (14) are consistent with the Itô–Girsanov expression for the Radon–Nikodym (RN) derivative when considering continuous-time Brownian processes. We attach Itô’s name to this expression to emphasize the use of Itô’s lemma to treat the stochastic integral in the standard Girsanov expression. As indicated in Appendix A, the RN derivative is necessary to eliminate divergences when taking the continuous-time limit. The equations for the common-path construction are valid only when all possible endpoints are included. In Section 3.2, we examine the case where the endpoint is constrained. With such bridge paths, we find a different result. Below, in Section 3.1, for two examples, we determine the KL divergence for the common-path construction and obtain nonzero results.

## 3. Results

In this section, we present two illustrative examples that demonstrate that the common-noise construction produces both the physical continuous-time limit of the change of measure and the KL divergence between two Brownian processes. Furthermore, we investigate the prevalent practice of sampling transition paths by exclusively using bridge paths. When using such a restricted subset of paths, Jensen’s inequality is violated. Thus, such sampling methods lack a sound mathematical foundation.

### 3.1. The Physical Limit

Here, we show two examples where it is clear that the common-noise construction produces the physical continuous-time limit. As noted above, if one uses the common-noise procedure to generate the KL divergence, then one finds that it vanishes. First, we examine the change of measure as one switches from free Brownian motion to a process with constant drift. Consider discrete-time processes $Q_{c}$ and $Q_{0}$ corresponding to

(16) $$Q_{c}:\;\;x_{i}-x_{i-1}\;-\;c\;\Delta t\;=\;\sqrt{2\;\epsilon \;\Delta t\;}\;\eta_{i}\;\;\;\mathrm{and}$$

(17) $$Q_{0}:\;\;z_{i}-z_{i-1}\;=\;\sqrt{2\;\epsilon \;\Delta t\;}\;{\tilde{\eta }}_{i}\;.\;\;\;$$

We see that $x_{t}\;=\;z_{t}\;+\;c\;t$ defines the bijective map that links path pairs with a common-noise history ($\eta_{i}={\tilde{\eta }}_{i}$), with $x_{0}=z_{0}=0$, and $x_{T}=z_{T}\;+\;c\;T$. The value of $D_{KL}(Q_{0}\;||\;Q_{c})$ vanishes. Now consider the Fokker–Planck equation for the process $Q_{c}$, which is given by

(18) $$\frac{\partial }{\partial t}\;\rho \left(x,\;t\right)\;=\;-\;c\;\frac{\partial }{\partial x}\;\rho \left(x,\;t\right)\;+\;\epsilon \;\frac{\partial^{2}}{\partial x^{2}}\rho \left(x,\;t\right)\;.$$

Making the substitution x=z+ct, Equation (18) reduces to the diffusion equation for the shifted probability function. Thus, the solution to the Fokker–Planck equation for the process $Q_{c}$ is consistent with this bijective map.

However, here we show that if one uses the common-path procedure, one finds the KL divergence to be positive and thus the measures are not identical. Note that for the common-path construction, in Equations (16) and (17), $\eta_{i}\neq {\tilde{\eta }}_{i}$. We use the processes $Q_{c}$ and $Q_{0}$ defined above and consider Equation (12), and note that J vanishes when the external force is zero. We generate paths *Z* that correspond to free Brownian motion (zero force). Then the path *Z* is inserted into $J_{N_{t},\;c}\left(Z\right)$ for the process $Q_{c}$. Thus, ${\tilde{D}}_{KL}^{CT}(Q_{0}\;||\;Q_{c})$ is given by

(19) $${\tilde{D}}_{KL}^{CT}(Q_{0}\;||\;Q_{c})\;=\;E_{0}\left(\frac{1}{2\;\epsilon }\int_{0}^{T}\frac{1}{2}\;c^{2}\;dt\right)\;=\frac{1}{4\;\epsilon }\;c^{2}\;T$$

where *T* is the (time) length of the path, $E_{0}\left(..\right)$ is the expected value of (..) for the ensemble of free Brownian paths starting at the origin, and $z_{0}\;=\;0$, $E_{0}\;z_{t}\;=0$, and $E_{0}\;z_{t}^{2}\;=2\;\epsilon \;t$. Within the common-path construction, the expression for ${\tilde{D}}_{KL}^{CT}(Q_{0}\;||\;Q_{c})$ is positive when *c* is nonzero and *T* is positive.

The result in Equation (19) demonstrates that, in the continuous-time limit, the two constructions produce different results for the KL divergence. Since in the continuous-time limit, Δt never becomes zero but only approaches it, neither construction produces the KL divergence for continuous-time Brownian processes. Clearly, the continuous-time limit depends on the manner in which the KL divergence is constructed.

Now, consider the Ornstein–Uhlenbeck (OU) process. The discrete-time OU process is given by

(20) $$Q_{OU}:\;\;x_{i}-x_{i-1}\;+A\;x_{i-1}\;\Delta t\;=\;\sqrt{2\;\epsilon \;\Delta t\;}\;\eta_{i}\;,$$

with A>0 being the OU constant. By combining Equations (17) and (20), we see that the common-noise construction implies that

(21) $$x_{i}-x_{i-1}\;+A\;x_{i-1}\;\Delta t\;=\;z_{i}-z_{i-1}\;.$$

Using $y_{t}=\exp\;\left(A\;t\right)\;x_{t}$, one is able to generate the analytic solution to the continuous-time OU process. With the continuous-time limit of Equation (21), we arrive at

(22) $$dx_{t}\;+A\;x_{t}\;dt=\exp\;\left(-A\;t\right)\;dy_{t}=dz_{t}=\sqrt{2\;\epsilon }\;dW_{t}.$$

and thus

(23) $$y_{t}\;=\;y_{0}\;+\;\sqrt{2\;\epsilon \;}\;\int_{0}^{t}\exp\;\left(A\;s\right)\;dW_{s}\;.$$

This shows that the analytical solution is consistent with the common-noise construction with a vanishing KL divergence. On the other hand, using Itô calculus, the common-path construction uses produces non-vanishing KL divergences, namely

(24) $$\begin{array}{c} {\tilde{D}}_{KL}(Q_{0}\;||Q_{OU})\;=\;\frac{1}{4}A^{2}\;T^{2}\;\;\mathrm{and}\\ {\tilde{D}}_{KL}(Q_{OU}\;||Q_{0})\;=\frac{1}{8}\left(\exp\;(-2\;A\;T)-\;1\;+2\;A\;T\right). \end{array}$$

### 3.2. Bridge Paths

In this section, we examine the common-path construction when the paths are constrained to start and end at the origin, $z_{0}\;=\;z_{T}\;=\;0$. In particular, we consider Brownian Bridge and the OU bridge processes. Here, we look at ${\tilde{D}}_{KL}^{CT}(Q_{0}^{BB}\;||\;Q_{OU}^{B})$, which becomes

(25) $$\begin{array}{c} {\tilde{D}}_{KL}^{CT}(Q_{0}^{BB}\;||\;Q_{OU}^{B})\;=E_{0}^{BB}\;J_{N_{t},\;OU}^{B}\left(Z\right)\;\\ =\;E_{0}^{BB}\;\left(\;\frac{1}{2\;\epsilon }\int_{0}^{T}\;\;dt\;\left(\;\frac{A^{2}}{2}z_{t}^{2}-\epsilon \;A\;\right)\;\right)\;, \end{array}$$

where the symbols $Q_{0}^{BB}$ and $Q_{OU}^{B}$ denote the Brownian Bridge and the OU bridge processes, respectively, and $E_{0}^{BB}\;\left(..\right)$ denotes the expected value of (..) generated by the Brownian Bridge process. The time-dependent variance of Brownian Bridges is $E_{0}^{BB}\;x_{t}^{2}\;=2\;\epsilon \;t\;\left(1-t/T\right)\;.$ Thus, we find that

(26) $$\begin{array}{c} {\tilde{D}}_{KL}^{CT}(0_{BB}\;||\;A_{OUB})=\frac{A\;T}{12}\left(A\;T\;-\;6\right)\;. \end{array}$$

This result indicates that something is amiss, as the KL divergence is to be non-negative, which is not the case when AT<6. Equation (26) represents a violation of Jensen’s inequality. The source of this odd result lies in the truncation of the path space caused by the ending constraint. Paths that start at the origin will have (infinitely) many possible endings. The percentage of paths that end near the origin will depend on the underlying potential. This then shows that it is not correct to use the common-path construction to calculate the KL divergence for measures associated with doubly constrained paths, so-called bridge paths. Furthermore, similar considerations exist in interpreting results generated by reweighting schemes [12,13], as bridge paths are used.

## 4. Discussion

In this section, we delve into the results presented in Section 3. Firstly, we address numerical discrepancies that distinguish the results obtained using the common-path construction from those obtained using the common-noise construction. Subsequently, we explore the concept of “thermodynamic action” and show that it is a mathematical artifact.

### 4.1. Numerical Details

We now examine the implications for the KL divergence of using the common-noise construction, Equation (10), or using the common-path construction, given by Equation (15). In Equation (10), we use the following equality,

(27) $$\begin{array}{c} \sum_{i=1}^{N_{t}}{\left(\left(x_{i}-x_{i-1}\right)-\Delta t\;f_{1}\left(x_{i-1}\right)\right)}^{2}\;\\ \;=\;\sum_{i=1}^{N_{t}}{\left(\left(z_{i}-z_{i-1}\right)-\Delta t\;f_{2}\left(z_{i-1}\right)\right)}^{2}\;. \end{array}$$

The above implies that

(28) $$\begin{array}{c} \sum_{i=1}^{N_{t}}\;\left({\left(x_{i}-x_{i-1}\right)}^{2}\;-\;{\left(z_{i}-z_{i-1}\right)}^{2}\right)\\ \;=\;O(\Delta t)+\mathrm{higher}\mathrm{order}\mathrm{terms}. \end{array}$$

Consider the continuous-time limit of the quadratic variation, namely,

$${\left[\;X\;\right]}_{T}\;=\lim_{\Delta t\to \;0}\;\sum_{i=1}^{T/\Delta t}{\left(x_{i}-x_{i-1}\right)}^{2}\;,$$

Then, ${\left[\;X\;\right]}_{T}\;=\;{\left[\;Z\;\right]}_{T}\;=\;2\;\epsilon \;T$ almost surely. By reordering the sum, one may choose to use the continuous-time limits of the quadratic variation to eliminate all the terms on the left-hand side of Equation (28). However, the quantity described in Equation (10) does not vanish in the continuous-time limit as

(29) $$\begin{array}{c} \frac{1}{\Delta t}\sum_{i=1}^{N_{t}}\;\left({\left(x_{i}-x_{i-1}\right)}^{2}\;-\;{\left(z_{i}-z_{i-1}\right)}^{2}\right)\;\\ \;\;=\;O(\;1\;)+\mathrm{higher}\mathrm{order}\mathrm{terms}. \end{array}$$

Evidently, the common-path construction is consistent with the continuous-time limit of the quadratic variation being used in the calculation of the KL divergence. This lies in contrast to the common-noise construction where the continuous-time limit is taken only after the KL divergence is formed. Changing the order of arithmetic operations produces different results.

We have explored two constructions for the KL divergence for discrete-time Brownian processes. In the continuous-time limit, one construction produces a vanishing value for $D_{KL}$; the other produces ${\tilde{D}}_{KL}$, which looks similar to the Itô–Girsanov expression for the KL divergence and produces a non-vanishing value.

### 4.2. Thermodynamic Action

It is of great interest to understand how particles move from one free-energy basin to another and the size of the intervening energy barriers. Historically, path integrals gave an intuitive description of quantum mechanics [14]. There, one samples all doubly constrained paths to determine the transition amplitude. The similarity of the diffusion equation and Schrödinger equation inspired the use of the OM function as a “thermodynamic action” to investigate barrier hopping transitions driven by Brownian dynamics. The OM functional (Equation (4)) was modified in the 1970s to use the continuous-time limit using the Itô–Girsanov (IG) expression of the Radon–Nikodym derivative [8,15,16,17,18,19,20,21,22,23,24,25,26,27]. In particular, this modified version of the OM functional $I^{IG}$ is given by

(30) $$\begin{array}{c} I^{IG}=\frac{\Delta t}{2\epsilon }\;\sum_{i=1}^{N_{t}}\;\left\{\frac{1}{2}\;{\left(\frac{x_{i}-x_{i-1}}{\Delta t}\right)}^{2}\;+\;G\left(x_{i-1}\right)\right\}\\ \;\;\Rightarrow \frac{1}{2\epsilon }\int_{0}^{T}\;\;dt\;\left\{\frac{1}{2}{\left(\frac{dx_{t}}{dt}\right)}^{2}\;+\;G\left(x_{t}\right)\right\}\;, \end{array}$$

where *G* is the path potential and is defined (in one dimension) as

(31) $$G\left(x\right)=\frac{1}{2}\;f^{2}\left(x\right)-\epsilon \;U^{\prime \prime }\left(x\right)\;.$$

This form of the OM functional has been in methods for sampling rare events by constructing doubly pinned diffusion paths [16,28,29,30,31,32,33]. Graham [16] and Eyink [34] used the “least-action” principle to discuss the minimization of the transformed OU functional as a “thermodynamic action”, as the value of $\int \;dt\;G(x_{t})$ depends on the path.

As previously pointed out [6], Equations (3) and (4) show that the OM functional is independent of the path. The values of the OM functional are Gaussian-distributed with the mean and variance being $N_{t}/2$. Any path dependence would be a consequence of correlation between the noise and the particle positions. Such correlations are unphysical; the noise is a property of the thermal bath and is independent of the system being studied. Looking at the form of Equations (30) and (31), the minimum of $I^{IG}$ can be dominated by the maximum of G(x). Such maxima can lead to MPPs (most probable paths) that are unphysical [35] or lead to an unphysical ensemble of paths when using Hybrid Monte Carlo sampling methods [5,6,7]. By using Equations (30) and (31), one has introduced an uncontrollable approximation.

Now, consider the method known as MinActionPath, which was introduced in the paper [36]. This method is employed to calculate the details of molecular transformations. It involves approximating the potential energy in multiple free-energy basins using quadratic polynomials. The fundamental principle behind this approach is that since the Laplacian is a constant, it can be eliminated from Equation (31). However, it is important to recognize that for each well the constant will be different. Taking this difference into account makes the minimization of the OM functional over a specific time interval much more complex. Additionally, this method ignores the role of saddle points. This method introduce additional uncontrolled approximations. Contrary to the comment in the work of Carter et al. [37], calculations based on MinActionPath have little to say about the physical meaning of the minimizer of the Itô–Girsanov expression.

We turn to compare classical path integrals with their quantum counterparts, as used in the path-integral formulation of quantum mechanics [14]. When considering paths with fixed endpoints, the minimization of the quantum action determines the physical properties of such paths. This contrasts with the “thermodynamic action”, which is a property of the noise transmitted from the thermal reservoir and is independent of the path. Typically, the Wick rotation (from imaginary time to inverse temperature) is used to connect quantum mechanics to statistical mechanics. In this case, blindly applying the Wick rotation would obscure this distinction.

Even though the noise does not contain information about the system, paths generated in discrete time are not without thermodynamic information. For ergodic systems, the equivalence of the microcanonical and canonical ensembles allows one to extract such information. In particular, consider a particle moving in a system with two free-energy basins, *A* and *B*. For long paths, the times the particle spends in either basins, $t_{A}$ and $t_{B}$, are related to the free-energy difference $F_{A}-F_{B}$ by

(32) $$F_{B}-F_{A}=-\epsilon \ln\left(\frac{t_{B}}{t_{A}}\right).$$

Using an ensemble of shorter (time) doubly constrained paths, the free-energy differences can be extracted in a similar manner [7]. The form of $I^{IG}$, Equation (30), was derived using continuous-time Brownian processes. As shown above, for discrete-time processes, the use of the Itô–Girsanov expression for the Radon–Nikodym derivative leads to a change of measure with a nonzero KL divergence, and leads to an uncontrolled approximation to the underlying measure. Computer algorithms are based on discrete-time processes, where such an uncontrolled approximation can lead to unphysical results.

Now, consider the frequency dependence of the (time) Fourier transform of the Brownian paths. Above some finite frequency cutoff, the high-frequency components of the path are independent of the force (drift), as the potential (and the force) must be smooth. The path multiplicity depends exponentially on the number of frequency components. As the time increment, Δt, approaches zero, the logarithm of the multiplicity diverges. One can make an analogy with the standard physical interpretation of thermodynamics. To do so, we use the Boltzmann definition of the Entropy as the logarithm of the (path) multiplicity, and equate the energy with the cost function, the logarithm of the probability, here given in Equation (4). For smooth paths, only a relatively small number of frequency components are nonzero. As one decreases the time step, Δt, of the numerical representation of Brownian paths, the number of frequency components increases. However, when comparing the paths for any nonzero Δt to those of a continuous-time process, one finds that an infinite number of frequency components are discarded. The multiplicity of paths grows exponentially as Δt decreases. For coarse time grids, the multiplicity, and thus the Entropy, is small, and the distribution of paths is dominated by the most probable ones. The multiplicity increases exponentially as the time grid becomes finer; the cost function becomes less important.

Remember that the origin of the OM functional is based on Equation (3), which only depends on the number of Gaussian random variables; this functional is independent of the prefactor of the Weiner function in Equation (1). Thus, the “small-noise” limit of Huang, Huang, and Duan [38] does not have any physical meaning. Finally, in continuous-time processes, the multiplicity and the Entropy diverge and, concomitantly, the cost function becomes irrelevant. Thus, all paths generated by continuous-time Brownian processes are equally likely, independent of the drift, the deterministic component of the force.

We conclude that the use of Equation (30) for the OM functional is troublesome. If used as the basis of computer algorithms, unphysical results may be generated. The path integral over $G(x_{t})$ is only correct if one uses paths generated by continuous-time processes. But for continuous-time processes, $dx_{t}/dt$ is not defined. If one uses discrete-time processes, $dx_{t}/dt$ can be approximated, but the use of $G(x_{t})$ is an uncontrolled approximation, as shown in the above sections.

## 5. Conclusions

In this analysis, we examined the KL divergence, denoted as $D_{KL}$, between two discrete-time Brownian processes and then at the limit as Δt approaches zero. We discovered that the KL divergence vanishes only when we consider common-noise histories. However, when we consider ${\tilde{D}}_{KL}$ derived from the common-path construction, we obtain a formula that reduces, in the continuous-time limit, to one that resembles the Itô–Girsanov expression for the Radon–Nikodym derivative, and does not vanish. On the other hand, all continuous-time Brownian processes have equivalent measures.

How do we reconcile these results? The limit map is not commutative. How the limit is taken matters. Analyzing the continuous-time processes produces a result different from the procedure of taking the limit of discrete-time processes as the value of the time step vanishes. It is evident that the results obtained using the common-noise construction align with physical considerations, as demonstrated by the two examples presented in Section 3.1

The two limiting processes differ. For discrete-time processes, as shown in Section 3.1, the physical limit must be the one that employs the common-noise construction to create path pairs, as the KL divergence vanishes for any value of Δt>0 no matter how small Δt becomes. Remember that in such a limiting process, Δt never becomes zero; continuous-time processes are never considered. This is an example of what Berry [39] called a “singular limit”.

This work has significant ramifications in numerous domains. We consider two. First, consider computer algorithms that aim to explore transition pathways by numerically generating an ensemble of paths that are constrained to start in one free-energy basin and end in another. If one uses the Itô–Girsanov expression for the Radon–Nikodym derivative, and uses a nonzero time step, one has introduced an uncontrolled approximation. The resulting computer simulations may become widely unphysical, as shown previously [5,6,7].

A second byproduct of the current work is as follows: the OM functional (Equation (4)) cannot be the thermodynamic action [17] that can be minimized, and the MPP, most probable path [8,40], is unphysical; it is simply a mathematical artifact. The OM functional is a property of the noise and has a mean and variance independent of the path. When the functional is expressed in terms of the path positions, and when the Itô–Girsanov expression is used, the functional only seems to have minima. However, as indicated above, the infinite multiplicity for continuous-time processes relegates all extrema to a set of measure zero. In numerical approaches, one may try to compensate for the structure of the “cost” function by decreasing the size of Δt. Even with the modern Hybrid Monte Carlo methods [7,41] that have a dimension-free acceptance rate, this presents a computational hurdle since the size of the phase space increases exponentially as the size of Δt decreases.

All thermodynamically allowed paths are equally probable. It is the multiplicity of paths that determines what reaction pathways dominate. Simply put, more paths pass over small barriers than over large ones. More paths pass through broad free-energy valleys than through narrow ones. Additionally, processes described by discrete time steps are significantly different from continuous-time processes, as in the former, no matter how small Δt is, an infinite number of frequency components have been discarded. Finally, consider doubly constrained paths: all paths starting at $x_{A}$ and, after a time *T*, ending at $x_{B}$. For quantum path integrals, the extremum of the quantum action governs the physical trajectory of the particle as it transverse from $x_{A}$ to $x_{B}$. In contrast, for classical path integrals, the “thermodynamic action” yields a value that is independent of the endpoints. This distinction would not be discernible by merely applying the Wick rotation.

We now state an observation based on the following: (1) the continuous-time limit of the quadratic variation does not depend on the drift, (2) for small values of Δt, the noise term dominates the drift term in Equation (2), (3) for continuous-time Brownian processes, the drift can be transformed away, and (4) the multiplicity of paths diverges in the limit Δt→0. Thus, the paths generated by continuous-time Brownian processes are independent of the drift, as all such paths correspond to the Gaussian process with zero drift. For continuous-time processes, the random buffeting due to the noise overwhelms the effects of the deterministic force. The change of measure for continuous-time processes is given in terms of the Radon–Nikodym derivative, which differs from the value for the continuous-time limit of discrete-time processes.

With apologies to P.W. Anderson [42], when it comes to the high-frequency modes of Brownian paths, the statement “Infinitely more is singularly different” holds true.

## Data Availability

The original contributions presented in this study are included in the article. Further inquiries can be directed to the corresponding author.

## Appendix A. Radon–Nikodym Derivative and the KL Divergence: A Scalar Example

In this section, we present a scalar example of the Kullback–Leibler (KL) divergence and its relationship with the change of measure. That the KL divergence is non-negative ($D_{KL}\geq 0$) is due to Jensen’s theorem for convex functions [43].

To begin, consider two potentials, scalar functions defined on the real numbers

(A1) $$\begin{array}{c} U_{2}\left(x\right)=\frac{1}{2}\;A\;x^{2}\;\mathrm{and}\;U_{8}\left(x\right)=\frac{1}{8}\;x^{8}, \end{array}$$

and the corresponding (Boltzmann) probability distributions, defined as

(A2) $$\begin{array}{c} P\left(x\right)=\frac{1}{Z_{2}}\exp\;\left(-U_{2}\left(x\right)/\epsilon \right) \end{array}$$

(A3) $$\begin{array}{c} Q\left(x\right)=\frac{1}{Z_{8}}\exp\;\left(-U_{8}\left(x\right)/\epsilon \right). \end{array}$$

Defining L(x) to be the equivalent of the Radon–Nikodym derivative, the change of measure is defined as

(A4) $$\begin{array}{c} L\left(x\right)=\frac{Q(x)}{P(x)}\;\;\;\mathrm{with}\;\;\;E_{Q}\;1=E_{P}\;L\left(x\right)=1, \end{array}$$

with i=P,Q and $E_{i}\left(..\right)$ being the expected value of (..) using the probability distribution given in Equation (A2). Then, the KL divergence is given by

(A5) $$\begin{array}{c} D_{KL}\left(P||Q\right)=-E_{P}\;\ln\frac{Q(x)}{P(x)}\\ \;=\ln Z_{8}-\ln Z_{2}+\frac{1}{\epsilon }\;E_{P}\left(U_{8}\left(x\right)-U_{2}\left(x\right)\right). \end{array}$$

To relate the KL divergence to physics quantities, we define Φ as

(A6) $$\begin{array}{c} \Phi =-\epsilon \ln Z_{2}\;+\;E_{P}\left(U_{8}\left(x\right)-U_{2}\left(x\right)\right)\\ \;=-\epsilon \ln Z_{8}+\epsilon \;D_{KL}\left(P||Q\right), \end{array}$$

with $Z_{2}=\sqrt{2\;\pi \;\epsilon /A}$, $Z_{8}=\;2^{11/8}\;\epsilon^{1/8}\;\Gamma \left[9/8\right]$, $E_{P}\;U_{2}\left(x\right)=\epsilon /2$, and $E_{P}\;U_{8}\left(x\right)=\;{\left(\epsilon /A\right)}^{4}\;105/8$. This then shows the equivalence of the inequality $D_{KL}\left(P||Q\right)\geq 0$ with $\Phi \geq -\epsilon \ln Z_{8}$. This latter expression is the Gibbs–Bogoliubov inequality [44], which can be used to generate a bound to the free energy.

The ratio L defined in Equation (A4) becomes the Radon–Nikodym derivative in the theory of continuous-time Brownian processes. Unlike the ratio, the numerator and denominator are not well defined for such processes. However, the ratio is well defined, is called the Radon–Nikodym derivative, and is usually denoted as dQ/dP.

## Appendix B. Use of Itô Calculus

In this section, we explore how Itô’s lemma is used in the Radon–Nikodym derivative. The starting point is the expansion of the potential energy function,

(A7) $$dU=U^{\prime }\left(x\right)\;dx\;+\;\frac{1}{2}\;U^{\prime \prime }\left(x\right)\;dx^{2},$$

with $U^{\prime }\left(x\right)$ and $U^{\prime \prime }\left(x\right)$ being the first and second derivatives of U(x). The next step is to use dx from the SDE, Equation (1), and insert it into the last term of Equation (A7). We arrive at the low-order expansion

(A8) $$\begin{array}{c} dU_{t}=-f\left(x_{t}\right)\;dx\;+\;\frac{1}{2}\;U^{\prime \prime }\left(x_{t}\right)\;{\left(f\left(x_{t}\right)\;dt\;+\;\sqrt{2\;\epsilon \;}\;dW_{t}\right)}^{2}\\ \;\;\approx -f\left(x_{t}\right)\;dx\;+\epsilon \;U^{\prime \prime }\left(x_{t}\right)\;dt\;. \end{array}$$

Then, integrating dU over a segment of a path, with $x_{t}=x_{0}$ at $t=t_{0}$ and $x_{t}=x_{1}$ at $t=t_{1}$, we obtain

(A9) $$\begin{array}{c} -\;\int_{x_{0}}^{x_{1}}\;\;\;\;f\left(x_{t}\right)\;dx=U\left(x_{1}\right)-U\left(x_{0}\right)-\epsilon \int_{t_{0}}^{t_{1}}\;\;U^{\prime \prime }\left(x_{t}\right)\;dt.\; \end{array}$$

The above equation is sometimes called the “integration-by-parts” rule for Itô calculus. The left-hand side of Equation (A9) is recognizable as the continuous-time limit of the last term in $J_{N_{t},\;\alpha }$, as defined in Equation (12).
